# BiC2PAM: constraint-guided biclustering for biological data analysis with domain knowledge

**DOI:** 10.1186/s13015-016-0085-5

**Published:** 2016-09-14

**Authors:** Rui Henriques, Sara C. Madeira

**Affiliations:** INESC-ID and Instituto Superior Técnico, Universidade de Lisboa, Lisbon, Portugal

## Abstract

**Background:**

Biclustering has been largely used in biological data analysis, enabling the discovery of putative functional modules from omic and network data. Despite the recognized importance of incorporating domain knowledge to guide biclustering and guarantee a focus on relevant and non-trivial biclusters, this possibility has not yet been comprehensively addressed. This results from the fact that the majority of existing algorithms are only able to deliver sub-optimal solutions with restrictive assumptions on the structure, coherency and quality of biclustering solutions, thus preventing the up-front satisfaction of knowledge-driven constraints. Interestingly, in recent years, a clearer understanding of the synergies between pattern mining and biclustering gave rise to a new class of algorithms, termed as pattern-based biclustering algorithms. These algorithms, able to efficiently discover flexible biclustering solutions with optimality guarantees, are thus positioned as good candidates for knowledge incorporation. In this context, this work aims to bridge the current lack of solid views on the use of background knowledge to guide (pattern-based) biclustering tasks.

**Methods:**

This work extends (pattern-based) biclustering algorithms to guarantee the satisfiability of constraints derived from background knowledge and to effectively explore efficiency gains from their incorporation. In this context, we first show the relevance of constraints with succinct, (anti-)monotone and convertible properties for the analysis of expression data and biological networks. We further show how pattern-based biclustering algorithms can be adapted to effectively prune of the search space in the presence of such constraints, as well as be guided in the presence of biological annotations. Relying on these contributions, we propose BiClustering with Constraints using PAttern Mining (BiC2PAM), an extension of BicPAM and BicNET biclustering algorithms.

**Results:**

Experimental results on biological data demonstrate the importance of incorporating knowledge within biclustering to foster efficiency and enable the discovery of non-trivial biclusters with heightened biological relevance.

**Conclusions:**

This work provides the first comprehensive view and sound algorithm for biclustering biological data with constraints derived from user expectations, knowledge repositories and/or literature.

## Introduction

Biological data are characterized by the presence of local patterns, whose discovery has been widely studied and motivated in the context of biclustering [1, 2]. In particular, the relevance of biclustering has been largely shown in the analysis of gene expression data (to discover transcriptional modules described by subsets of genes correlated in subsets of samples [2]) and biological networks (to unravel meaningfully dense regions from weighted adjacency matrices derived from interaction data [3]). A key question in the field of biclustering is how to benefit from the increasingly available domain knowledge. Initial attempts to incorporate background knowledge from user expectations [4–6] and knowledge-based repositories [7–10] within biclustering showed its importance to explore efficiency gains and guarantee relevant solutions. However, these attempts only support very specific forms of knowledge and cannot be extended to flexibly constrain the desirable properties of outputted biclusters. Furthermore, due to the complexity of the biclustering task^1^, most of the existing algorithms: (1) are based on greedy or stochastic approaches, producing sub-optimal solutions; and (2) usually place restrictions on the allowed structure, coherency and quality of biclusters, compromising the flexibility of the outputs [2, 11]. In this context, these biclustering approaches cannot be extended to incorporate knowledge-driven constraints since their restrictions may a priori contradict the inputted constraints.

Recent attempts to perform biclustering based on enhanced pattern mining searches [8, 12, 13], termed as pattern-based biclustering, showed the unprecedented possibility to efficiently discover arbitrarily positioned biclusters with parameterizable size, coherency and quality [2, 14]. In this context, two valuable synergies can be identified between pattern-based biclustering and knowledge incorporation. First, the optimality and flexibility of pattern-based biclustering solutions provide an adequate basis upon which knowledge-driven constraints can be incorporated. Pattern-based biclustering tackles the restrictions of peer algorithms, being an adequate candidate to flexibly constrain the desirable properties of the target solution space. Second, the effective use of domain knowledge to guide pattern mining searches has been largely studied in the context of domain-driven pattern mining [15, 16].

Despite these synergies, two major problems persist. First, there is a lack of understanding on whether domain-driven pattern mining and biclustering can be consistently integrated. In particular, there is not a solid ground on how to map the commonly available background knowledge in the form of constraints to guide the biclustering task. Second, pattern-based biclustering algorithms depend on a specific variant of pattern mining, referred as full-pattern mining, which has been scarcely studied in the context of domain-driven pattern mining. In fact, although new full-pattern mining searches have been recently proposed to guarantee the scalability of the biclustering task over large and dense data [17, 18], there are not yet contributions on how these searches can be adapted to incorporate background knowledge.

This work addresses these problems. To this end, it extends pattern-based biclustering algorithms using principles from domain-driven pattern mining to seize large efficiency gains in the presence of background knowledge. Furthermore, it shows how functional annotations and constraints with succinct, (anti-)monotone and convertible properties can be used to guide the biclustering task. The major contributions are fivefold:

- integrative view of domain-driven pattern mining and (pattern-based) biclustering. The consistency of this view is shown for patterns given by frequent itemsets, association rules and sequences;
- principles for biclustering tabular data in the presence of an arbitrary number of annotations per observation (derived from knowledge repositories and literature);
- list of meaningful constraints with succinct, (anti-)monotone and convertible properties for biological data contexts with a focus on gene expression and network data;
- principles to specify, process and incorporate different types of constraints;
- extension of full-pattern miners based on pattern-growth searches to optimally explore efficiency gains from constraints with succinct, (anti-)monotone and convertible properties. In particular we show:
  - F2G [17] compliance with state-of-the-art pruning principles on pattern-trees;
  - IndexSpan [18] compliance with prefix-monotone checks on pattern-conditional data projections.

**Fig. 1:**
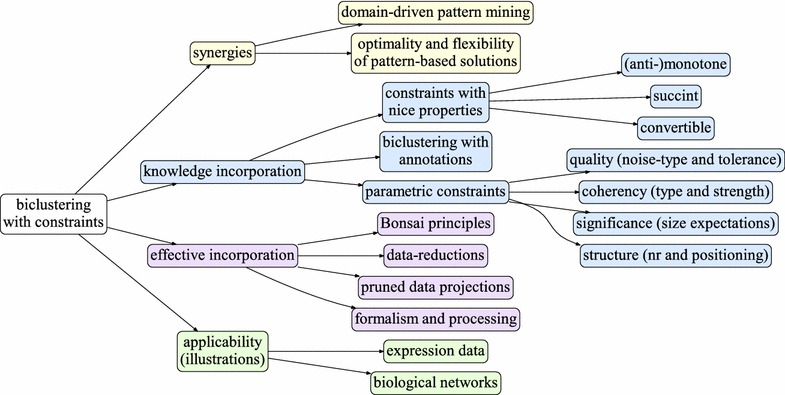
Proposed contributions to an effective incorporation of constraints with distinct properties into (pattern-based) biclustering tasks

Figure 1 provides a structured view on the proposed contributions and their applicability.

In this context, we propose BiClustering with Constraints using PAttern Mining (BiC2PAM), an algorithm that integrates recent breakthroughs on pattern-based biclustering [3, 14, 19, 20] and extends them to effectively incorporate constraints and annotations from domain knowledge.

Experimental results on synthetic and real data show the importance of incorporating background knowledge within pattern-based biclustering to seize large efficiency gains by adequately pruning the search space and to guarantee non-trivial and (biologically) relevant solutions.

This paper is structured as follows. First, we provide background on domain-driven pattern mining for pattern-based biclustering. Second, key contributions and limitations from related work are surveyed. Third, we list meaningful constraints in gene expression data and biological networks, and describe an algorithmic basis (BiC2PAM) for their incorporation. BiC2PAM is further extended to attain efficiency gains from constraints with nice properties. Fourth, we provide initial empirical evidence of BiC2PAM’s efficiency and ability to unravel non-trivial yet biologically significant biclusters. Finally, concluding remarks and major implications are synthesized.

## Background

### Biclustering, full-pattern mining and pattern-based biclustering

#### **Definition 1**

Given a real-valued matrix *A* with *n* rows* X* = $\{x_1,\ldots,x_n\}$ and* m* columns Y = $\{y_1,\ldots,y_m\}$, and elements $a_{ij}$ relating row $x_i$ and column $y_j$, the ***biclustering*** task aims to identify a set of biclusters $\{B_1,\ldots,B_p\}$, where each bicluster $B_k$ = $(I_k,J_k)$ is defined by a subset of rows $I_k\subset X$ and columns $J_k\subset Y$ satisfying specific criteria of homogeneity and statistical significance.

**Fig. 2:**
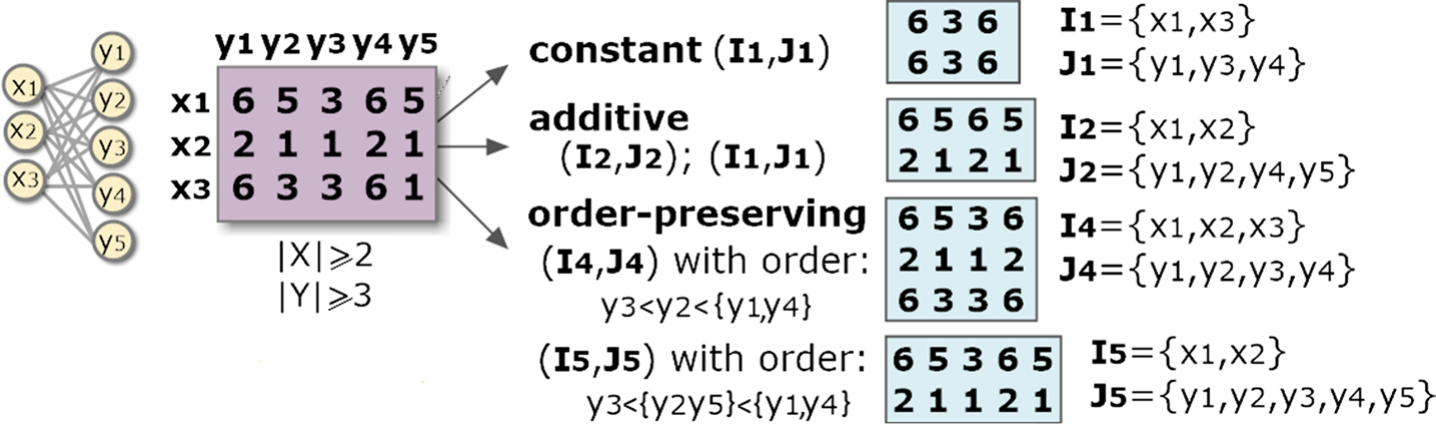
Pattern-based biclusters with distinct coherency assumptions

The *homogeneity* criteria determine the structure, coherency and quality of biclustering solutions, while the statistical significance of a bicluster determines whether its probability of occurrence deviates from expectations. The homogeneity of a biclustering model is commonly guaranteed through a *merit function*. Following Madeira’s taxonomy [2], existing biclustering algorithms can be grouped according to their homogeneity criteria (defined by the underlying *merit function*) and search paradigm (determining how the merit function is applied). The *structure* of a biclustering solution is essentially defined by the number, size and positioning of biclusters. Flexible structures are characterized by an arbitrary high set of (possibly overlapping) biclusters. The *coherency* of a bicluster is defined by the observed correlation of values (coherency assumption) and by the allowed deviation from expectations (coherency strength). A bicluster can have coherency of values across its rows, columns or overall elements, where the values typically follow constant, additive, symmetric and order-preserving assumptions [2]. Finally, the *quality* of a bicluster is defined by the type and amount of accommodated noise. Definitions 2 and 3 formalize these concepts, while Fig. 2 shows a set of biclusters with different coherencies in a symbolic dataset.

#### **Definition 2**

Let the elements in a bicluster $a_{ij}\in (I,J)$ have ***coherency across rows*** given by $a_{ij}$ = $k_j+\gamma _i+\eta _{ij}$, where $k_j$ is the expected value for column *j*, $\gamma _i$is the adjustment for row *i*, and $\eta _{ij}$ is the noise factor (affecting the ***quality*** of the bicluster). Let $\bar{A}$ be the amplitude of values in a matrix *A*. Given a matrix *A*, the ***coherency strength*** is a real value $\delta \in [0,\bar{A}]$, such that $a_{ij}=k_j+\gamma _i+\eta _{ij}$ where $\eta _{ij}\in [-\delta /2,\delta /2]$.

#### **Definition 3**

The $\gamma$ factors define the ***coherency assumption***: ***constant*** when $\gamma$ = 0, and ***additive*** otherwise. ***Symmetries*** can be accommodated on rows, $a_{ij}\times c_i$ where $c_i\in \{1,$ −$1\}$. ***Order***-preserving assumption is verified when the values of rows induce the same linear ordering across columns.

#### **Definition 4**

Given a bicluster *B* = (*I*, *J*), the bicluster ***pattern***$\varphi _{B}$ is given by the sequence of expected values ($k_j$) according to a permutation of columns in the absence of adjustments ($\gamma _i$ = 0) and noise ($\eta _{ij}$ = 0): $\{k_j \mid y_j\in J\}$, while its *support* is given by the number of rows satisfying the pattern: |*I*|.

Consider the additive bicluster (I,J) = ($\{x_1,x_2\}$,$\{y_1,y_2,y_3\}$) in $\mathbb {N}_0^+$ with coherency across rows. Assuming $x_{1}|J$= $\{1,3,2\}$ and $x_{2}|J$ = $\{3,4,2\}$, then this biclusters can be described by $a_{ij}$ = $k_j$ + $\gamma _i$ with the pattern $\varphi$ = {$k_1$ = 0, $k_2$ = 2, $k_3$ = 1}, supported by two rows with additive factors $\gamma _1$ = 1 and $\gamma _2$ = 3.

Despite the relevance of discovering optimal and flexible biclustering solutions to effectively incorporate knowledge-driven constraints, most of the existing biclustering algorithms are based on greedy or stochastic searches, producing sub-optimal solutions, and place restrictions (such as simplistic forms of coherency, fixed number of biclusters, non-overlapping structures) that prevent the flexibility of the outputs [2, 14].

*Pattern-based biclustering*. In recent years, a clearer understanding of the synergies between pattern mining and biclustering gave rise to a new class of algorithms, referred as pattern-based biclustering, aiming to address these limitations (no guarantees of optimality and flexibility). Pattern-based biclustering is inherently prepared to efficiently find exhaustive solutions of biclusters with the unprecedented possibility to customize their structure, coherency and quality. Such behavior explains why these algorithms are receiving an increasing attention for biological data analysis [3, 8, 12, 14, 19–21]. The major potentialities include: (1) efficient searches with optimality guarantees; (2) biclusters with flexible coherency strength and assumption [14, 19, 20]; (3) robustness to noise, missing values and discretization problems [14] by introducing the possibility to assign or impute multiple symbols to a single data element; (4) non-fixed number of biclusters arbitrarily positioned [12, 21]; (5) applicability to network data and sparse data matrices [3, 22]; among others.

At its core, pattern-based biclustering relies on the (iterative application of the) full-pattern mining task [14]. A full-pattern defines a region from the input data space, thus enclosing not only the underlying pattern (itemset, association rule, sequential pattern or graph with frequency and length above certain thresholds), but also its supporting rows and columns.

#### **Definition 5**

Let $\mathcal {L}$ be a finite set of items, and a pattern *P* to be a composition of items, either an itemset ($P\subseteq \mathcal {L}$), association rule ($P\,{:}\;P_1\rightarrow P_2$ where $P_1\subseteq \mathcal {L}\wedge P_2\subseteq \mathcal {L}$) or sequence ( *P* = $P_1\ldots P_n$ where $P_i\subseteq \mathcal {L}$). Let a ***transactional database****D*be a finite set of rows/transactions, each defining a composition of items. A transaction is commonly given by an itemset or sequence. Given *D*, let the coverage $\Phi _{P}$ of pattern *P* be the set of rows in *D* in which *P* is satisfied/occurs, and its ***support***$sup_P$ be the coverage size, $|\Phi _{P}|$. Let the ***length*** of a pattern |*P*| be the number of items.

#### **Definition 6**

Given a matrix *A*, let *D* be a transactional database derived from *A*: either the concatenation of items with their column index (transactions given by itemsets) or the ordering of column indexes according to the values per row (transactions given by sequences).* A****full-pattern*** is a tuple $(P,\Phi _{P},\psi _P,\Upsilon _P)$, where *P* is the pattern in *D*, $\Phi _{P}\subset X$ is its coverage (rows satisfying *P*), $\Psi _P\subset Y$ is the set of indexes (columns), and $\Upsilon _P$ is the original pattern in *A* (the corresponding itemset, rule or sequence prior to the concatenation or ordering of column indexes).

#### **Definition 7**

Given a matrix *A*, the mapped transactional database *D*, and a minimum support $\theta _1$ and pattern length $\theta _2$ thresholds, ***full-pattern mining*** consists of computing: $\{(P,\Phi _{P},\psi _P,\Upsilon _P) \mid sup_P \ge \theta _1\wedge |P|\ge \theta _2\}$.

**Fig. 3:**
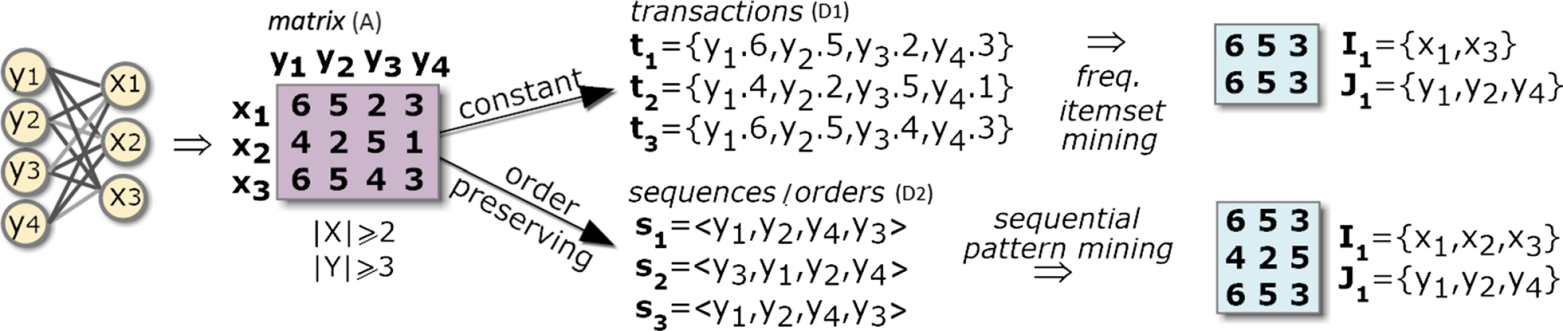
Discovery of biclusters with constant and order-preserving assumptions based on full-patterns (itemsets and sequences) discovered from transactional databases mapped from the original data matrix

Figure 3 shows how a symbolic matrix, *A*, is mapped into two distinct transactional databases (given either by index concatenations or orderings), $D_1$ and $D_2$, for the subsequent discovery of full-patterns. The concatenation of an item $\sigma \in \mathcal {L}$ with a column index in $y_i\in Y$ is represented as $y_i.\sigma$. The full-pattern in $D_1$ can be formally described as $(\{y_1.6,y_2.5,y_4.3\},\{t_1,t_3\},\{y_1,y_2,y_4\},$ 〈6, 5, 3〉).

Frequent itemsets can be discovered to compose constant, additive and multiplicative models [14]; sequential patterns are used to learn order-preserving models [19]; and rules can be composed to learn plaid models or tolerate parameterizable levels of localized noise [20]. Figure 3 further illustrates the paradigmatic cases where full-pattern mining is applied to discover constant and order-preserving biclusters.

In this context, the set of maximal biclusters (bicluster not contained in larger biclusters) are mapped from *closed full-patterns* (frequent yet not contained in larger patterns with same support). Definition 8 specifies the mapping between a full-pattern and a bicluster. For real-valued matrices, (real-valued) biclusters are mapped from full-patterns discovered under a parameterizable coherency strength ($$\delta$$$$\propto$$1/$$|\mathcal {L}|$$ where $$\mathcal {L}$$ is the discretization alphabet).

#### **Definition 8**

Given a transactional database *D* derived from a real-valued matrix, the set of ***maximal biclusters***$\cup _k (I_k,J_k)$ can be derived from the set of closed full-patterns $\cup _k P_k$ by mapping $I_k$ = $\Phi _{P_k}$ and $J_k$ = $\Psi _{P_k}$, where $\varphi _{B_k}$ = $\Upsilon _{P_k}$.

### Constraint-based biclustering

To formalize the task targeted in this work, we introduce below the concept of constraint in the context of biclustering, and further describe different types of constraints according to the selected full-pattern mining task.

A constraint is traditionally seen as a conjunction of relations (predicate) over a set of variables describing a given dataset [23]. Definitions 9 and 10 revise this notion to guarantee its proper applicability within (pattern-based) biclustering tasks.

#### **Definition 9**

In the context of pattern mining, a constraint is a predicate on the powerset of items $C{:}\;2^{\mathcal {L}}\rightarrow${true,false}. In the context of full-pattern mining, a ***full-constraint*** is a predicate on the powerset of original items, transactions, indexes and/or concatenations, $C\,{:}\;\{2^{\mathbf {Y}}\times 2^\mathcal {L},2^{\mathbf {X}},2^{\mathbf {Y}},2^{\mathcal {L}}\}\rightarrow${true,false}. A full-pattern $(P,\Phi _{P},\psi _P,\Upsilon _P)$ satisfies a full-constraint *C if*$C(P,\Phi _P,\psi _P,\Upsilon _P)$ is true.

#### **Definition 10**

A ***biclustering constraint*** is a predicate on a bicluster’s values per column, rows *I*, columns *J* and pattern $\varphi _B$, $C\,{:}\;\{2^{\mathbf {Y}}\times 2^\mathcal {L},2^{\mathbf {X}},2^{\mathbf {Y}},2^{\mathcal {L}}\}\rightarrow {true,false}.$ A bicluster *B* satisfies a constraint *C* if $C(\varphi _B\cdot J,I,J,\varphi _B)$ is true (or, alternatively, when the associated full-pattern satisfies a full-constraint).

Consider a matrix mapped into a transactional database with $$\mathcal {L}$$ = {a,b,c}. An illustrative full-constraint is $$y_1a\in P\wedge \{x_2,x_3\}$$$$\subseteq$$$$\Phi _P\wedge y_4$$$$\in$$$$\Psi _P\wedge \{b\}$$$$\subseteq$$$$\Upsilon _P$$, and the associated biclustering constraint is $$y_1a\in B \wedge \{x_2,x_3\}$$$$\subseteq$$$$I\wedge$$$$y_4\in J\wedge \{b\}$$$$\subseteq$$$$\varphi _B$$. Minimum support and minimum pattern length are the default full-constraints in full-pattern mining: $$C_{support}$$ = $$|\Phi _{P}|\ge \theta$$ and $$C_{length}$$ = $$|P|\ge \theta$$.

More interesting constraints with properties of interest include regular expressions or aggregate functions. In the presence of matrices with numeric or ordinal values, further constraints can be specified. In this context, a *cost table* is specified in addition to the alphabet of items (e.g. {a:0, b:1, c:2}). Depending on the type of full-pattern, multiple constraints can be applied against a cost table, including the paradigmatic cases of aggregate functions such as length, maximum, minimum, range, sum, mean and variance [24].

Some of these constraints are said to exhibit *nice properties* when their input can be effectively pushed deep into the pattern mining task [15] to prune the search space and therefore achieve efficiency gains. Below, we explore different types of constraints according to the selected full-pattern mining task for biclustering: itemset, rule-based and sequential-pattern constraints.

#### Itemset constraints

Regular expressions and aggregate functions are the most common form of constraints to guide frequent itemset mining. In this context, efficiency gains can be seized in the presence of constraints with succinct, (anti-)monotone and convertible properties.

##### **Definition 11**

Let $\mathcal {L}$ be a set of items and *P* be an itemset, $P\subseteq \mathcal {L}$. Let each item $\sigma \in \mathcal {L}$ have a correspondence with a real value, $c{:}\, \mathcal {L}\rightarrow \mathbb {R}$, according to a well-defined cost table. Let *v* be a real-valued constant and range(*P*) = *max*(*P*) − *min*(*P*), *max*(*P*) = $max\bigcup \nolimits _{_\sigma \in P}c(\sigma )$, *min*(*P*) = $min\bigcup \nolimits _{_\sigma \in P}c(\sigma )$ and *avg*(*P*) = $\sum \nolimits _{\sigma \in P}\frac{c(\sigma )}{|P|}$ be well-defined predicates. In this context:

- A constraint *C* is ***monotone*** if for any *P* satisfying *C*, *P* supersets satisfy *C* (e.g. $range(P)\ge v$).
- A constraint *C* is ***anti-monotone*** if for any *P* not satisfying *C*, *P* supersets do not satisfy *C* (e.g. $max(P)\le v$).
- Given a pattern $P'$ satisfying a constraint *C*, *C* is ***succint*** over *P* if *P* contains $P'$ (e.g. $min(P)\le v$).
- A constraint *C* is ***convertible*** with regards to an ordering of items $R_{\Sigma }$ if for any itemset *P* satisfying *C*, the *P* suffixes satisfy *C* or/and itemsets with *P* as suffix satisfy *C* (e.g. $avg(P)\ge v$).

To instantiate the formalized constraints, consider three observations ($$\mathbf {x}_1=\{a,b,c\}$$, $$\mathbf {x}_2=\{a,b,c,d\}$$, $$\mathbf {x}_3=\{a,d\}$$), a minimum support $$\theta _1$$ = 1 and length $$\theta _2$$ = 2, and the cost table {a:0, b:1, c:2, d:3}. The set of closed full-patterns satisfying: the monotone constraint *range*$$(P)\ge 2$$ is $$\{(\{a,b,c\},\{t_1,t_2\}),(\{a,d\},\{t_1,t_3\}),$$$$(\{b,d\},\{t_2\})\}$$; the anti-monotone constraint *sum*$$(P)\le 1$$ is $$\{(\{a,b\},\{t_1,t_2\})\}$$; the succint $$P\supseteq \{c,d\}$$ is $$\{(\{a,b,c,d\},\{t_2\})\}$$; and the convertible constraint *avg*$$(P)\ge 2$$ is $$\{(\{b,c,d\},\{t_2\})\}$$.

#### Association rule constraints

Constraints satisfying these properties can be also effectively applied in the context of association rule mining (for the discovery of noise-tolerant biclusters [1, 20]). In this context, constraints need to be satisfied by the antecedent, consequent, or can be alternatively applied during the generation of frequent itemsets, prior to the composition of rules.

Additional constraints to guarantee specific correlation/interestingness criteria [25] or the dissimilarity and minimality of rules [26] can be specified.

In the context of association rule-based biclustering, a full-constraint is evaluated against the union of items on the antecedent and consequent as well as the union of supporting transactions of the antecedent and consequent. Given $$P{:}\;P_1\rightarrow P_2$$ and a constraint *C*, *P* satisfies *C* if the full-pattern given by $$(\Upsilon _{P_1\cup P_2},\Phi _{P_1}\cup \Phi _{P_2},$$$$\psi _{P_1\cup P_2},P_1\cup P_2)$$ satisfies *C*.

#### Sequential pattern constraints

The introduced concepts can be further extended for the incorporation of constraints in the context of sequential pattern mining (for the discovery of order-preserving biclusters [19]). A sequence *P* is an ordered set of itemsets, each itemset being a set of indexes in *Y*. Given a matrix (*X*, *Y*) with *n* = 5 rows and *m* = 3 columns and a minimum support $\theta _1$ = 3, ($y_2\le y_1\wedge y_2\le y_3,\{x_2,x_4,x_5\},\{y_1,y_2,y_3\}$, $\langle y_2(y_1y_3) \rangle$) is an illustrative full-pattern. Interestingly, the sequential pattern $\Upsilon _{P}$ does not explicitly disclose the value expectations $\varphi _B$. Instead, $\Upsilon _{P}$ is associated with an ordering relation (such as $y_2\le y_1\wedge y_2\le y_3$). In this context, the following constraints can be specified: item constraints (e.g. $\{y_1,y_3\}\subseteq P$); length constraints (minimum/maximum number of precedences and/or co-occurrences); super-pattern constraints (patterns that contain a particular set of patterns as sub-patterns ${-}y_2\le y_1\subseteq P$); and, more interestingly, regular expressions (e.g. $P\equiv y_{\bullet }\le \{y_{\bullet },y_{\bullet }\}$). Constraints concerning value expectations can be also specified using the values from a given ordering based on the median of values from the supporting rows and columns (e.g. $b\le a$ or $1.3\le 0.4$). As a result, aggregate functions can be additionally specified within sequential pattern constraints.

With regards to properties of the aforementioned constraints: length constraints are anti-monotonic, while super-pattern constraints are monotonic. Item constraints, length constraints and super-pattern constraints are all succinct. Some aggregate constraints and regular expressions can also show nice properties [27].

## Related work

Related work is surveyed according to: (1) the contributions and limitations of existing attempts to perform biclustering with domain knowledge; (2) the state-of-the-art on domain-driven pattern mining; and (3) the existing efforts towards full-pattern mining and their adequacy to accommodate domain knowledge.

### Knowledge-driven biclustering

The use of domain knowledge to guide biclustering has been increasingly stressed since solutions with good homogeneity and statistical significance may not necessarily be biologically relevant. However, few biclustering algorithms are able to incorporate domain knowledge.

AI-ISA [7], GenMiner [8] and scatter biclustering [10] are able to annotate data with functional terms retrieved from repositories with ontologies and use these annotations to guide the search.

COBIC [28] is able to adjust its behavior (maximum-flow/minimum-cut parameters) in the presence of background knowledge. Similarly, the priors and architectures of generative biclustering algorithms [29] can also be parameterized to accommodate specific forms of background knowledge. However, COBIC and its generative peers support only the definition of constraints concerning the algorithm’s behavior and are not able to deliver flexible biclustering solutions.

Fang et al. [4] proposed a constraint-based algorithm enabling the discovery of dense biclusters associated with high-order combinations of single-nucleotide polymorphisms (SNPs). Data-Peeler [5], as well as algorithms from formal concept analysis [6] and bi-sets mining [30], are able to efficiently discover dense biclusters in binary matrices in the presence of (anti-)monotone constraints. However, these algorithms impose a very restrictive form of homogeneity in the delivered biclusters.

### Domain-driven pattern mining

A large number of studies explored how constraints can be used to guide pattern mining tasks. Two major paradigms are available: constraint-programming (CP) [16] and dedicated searches [15, 31]. CP allows pattern mining to be declaratively defined according to sets of constraints [16, 32]. These declarative models can allow for complex mathematical expressions on the set of full-patterns. Nevertheless, due to the poor scalability of CP methods, they have been only used in highly constrained settings, small-to-medium sized data, or to mine approximate patterns [16, 32].

Pattern mining searches have been adapted to seize efficiency gains from different types of constraints [15, 31, 33]. These efforts aim to replace naïve solutions based on post-filtering to guarantee the satisfaction of constraints. Instead, the constraints are pushed as deep as possible within the mining step for an optimal pruning of the search space. The nice properties exhibited by constraints, such as anti-monotone and succinct properties, have been initially seized in the context of frequent itemset mining by Apriori methods [31] to affect the generation of candidates. Convertible constraints can hardly be pushed in Apriori methods but can be adequately handled by pattern growth methods such as FP-Growth [15]. FICA, FICM, and more recently MCFPTree [15], are FP-Growth extensions to further explore opportunities from diverse constraints. The inclusion of monotone constraints is more complex. Filtering methods, such as ExAnte [34], are able to combine anti-monotone and monotone pruning based on reduction procedures. Empirical evidence shows that these reductions are optimally handled within pattern growth methods by adequately growing and pruning small FP-Trees (referred as FP-Bonsais) [33].

These contributions were extended for association rule mining [33, 35]. In particular, nice properties were studied for item constraints [35], support constraints [36], bounds interestingness criteria [37], and constraints on the structure and dissimilarity of rules (respectively referred as schema and opportunistic) [38].

Similarly, some studies proposed ways to effectively incorporate constraints within Apriori and pattern-growth searches for sequential pattern mining (SPM) [27, 39]. Apriori searches were first extended to incorporate temporal constraints and user-defined taxonomies [39]. Mining frequent episodes in a sequence of events [40] can also be viewed as a constrained SPM task by seeing episodes as constraints in the form of acyclic graphs. SPIRIT [41] revises the Apriori search to incorporate a broader range of constraints with nice properties and regular expressions. Pattern growth searches based on data projections, such as PrefixSpan, were only later extended by Pei et al. [27, 42] to support a wide-set of constraints with nice properties. Although multiple studies have been proposed on the use of temporal constraints for SPM, including length and gap constraints [27, 43], these constraints are not relevant for the aim of learning order-preserving models.

### Full-pattern mining with constraints

There are three major classes of full-pattern mining searches [1, 44, 45]: (1) AprioriTID-based searches, generally suffering from costs of candidate generation for dense datasets and low support thresholds; (2) searches with vertical projections, which show efficiency bottlenecks for data with a high number of transactions since the bitset cardinality becomes large and associated intersection procedures expensive; and (3) recently proposed pattern-growth searches based on the annotation of original pattern-growth structures with transactions’ identifiers. In particular, F2G [17] and IndexSpan [18] (default options in BicPAM, BiP, BicNET and BicSPAM biclustering algorithms [14, 19, 20, 22]) were the first pattern-growth searches for full-pattern mining aiming to surpass memory and time bottlenecks associated with bitset and diffset structures used by AprioriTID and vertical-based searches.

Despite the high number of contributions from domain-driven pattern mining, the ability of pattern-growth searches to effectively incorporate *full*-constraints with nice properties (Definition 9) was not yet demonstrated.

## Solution: Pattern-based biclustering with domain knowledge

This section extends pattern-based biclustering algorithms [1] to accommodate constraints by proposing BiC2PAM (BiClustering with Constraints using PAttern Mining). In what follows, we first provide principles for biclustering annotated biological data. Second, meaningful full-constraints with nice properties are listed to guide expression data analysis and network data analysis. The possibility to specify alternative constraints in order to customize the structure, coherency, quality and statistical significance of biclustering solutions according to available knowledge is discussed in Appendix. Third, we describe a set of principles for the specification, processing and incorporation of constraints within pattern-based biclustering. Finally, we adapt the full-pattern mining searches used within BiC2PAM in order to seize heightened efficiency gains by exploring the properties associated with the inputted constraints.

### Biclustering with annotations extracted from knowledge repositories and literature

Domain knowledge comes often in the form of annotations associated with specific rows and columns in a matrix (or nodes in a network). These annotations are often retrieved from knowledge repositories, semantic sources and/or literature. Annotations can be either directly derived from the properties associated with each row/column/node (e.g. properties of a gene or a sample in gene expression data) or can be implicitly predicted based on the observed values by using feature extraction procedures. For instance, consider the set of functional annotations associated with gene ontology (GO) terms [46]. A GO term is associated with an interrelated group of genes associated with a specific biological process. Since a gene can participate in multiple biological processes, genes can have an arbitrary number of functional annotations. As such, rows in an expression matrix (or nodes in a biological network) can be annotated with a non-fixed number of labels.

Pattern-based biclustering supports the integrated analysis of matrices and annotations recurring to one of two strategies. First, association rules or sequential rules can be used to guide the biclustering task in the presence of annotations according to the principles introduced by Martinez et al. [8]. In this context, annotations can either appear in the consequent, antecedent or on both sides of an association rule. Biclusters can then be inferred from these rules using the principles introduced by Henriques et al. [1]. Illustrating, a rule $\{y_12,y_42\}\rightarrow \{T_1, T_2\}$ supported by $\{x_1,x_3,x_5\}$ rows can be used to compose a bicluster $(\{y_1,y_4\},\{x_1,x_3,x_5\})$ with elements consistently associated with annotations $T_1$ and $T_2$. Learning association rules with levels of confidence (or alternative interestingness scores) below 100 % [20] is relevant to discover biclusters with consistent annotations without imposing a subset of annotations to appear on all rows/columns of each bicluster.

Second, the annotations can be included directly within data since pattern mining is able to rely on rows with an arbitrary length. To this aim, annotations are associated with a new dedicated symbol and appended to the respective rows, possibly leading to a set of observations with varying length. Consider the annotations $T_1$ and $T_2$ to be respectively associated with genes $\{x_1,x_3,x_4\}$ and $\{x_3,x_5\}$, an illustrative transactional database of itemsets for this scenario would be $\{x_1=\{a_{11},\ldots,a_{1m},T_1\},x_2=\{a_{21},\ldots,a_{2m}\},x_3=\{a_{31},\ldots,a_{3m},T_1,T_2\},\ldots\}$. Databases of sequences (for order-preserving biclustering) can be composed by appending terms either at the end or the beginning of each sequence.

Given these enriched databases, pattern mining can then be applied on top of these annotated transactions with succinct, (anti-)monotone and convertible constraints. Succinct constraints can be incorporated to guarantee the inclusion of certain terms (such as $$P\cap \{T_1,T_2\}$$$$\ne$$ 0). This is useful to discover, for instance, biclusters with genes participating in specific functions of interest. (Anti-)monotone convertible constraints can be, alternatively incorporated to guarantee, for instance, that a bicluster associated with a discovered pattern is functionally consistent, meaning that it can be mapped to a single annotation. The $$|P\cap \{T_1,T_2\}|\ge 1$$ constraint is anti-monotone and satisfies the convertible condition: if *P* satisfies *C*, the *P* suffixes also satisfy *C*.

Interestingly, the two previous strategies can be seen as equivalent when assuming that the discovery of the introduced class of association rules is guided by rule-based constraints and the discovery of patterns from annotated data is guided by itemset/sequence constraints.

### Biological constraints with properties of interest

Different types of constraints were introduced in Definition 11. In order to show how these constraints can be specified and instantiated, this section provides examples of meaningful constraints for gene expression and network data analysis.

Note that similar constraints can be formulated for the analysis of alternative biological data, including: structural genome variations to enable the discovery of high-order single-nucleotide polymorphisms; genome-wide data to find promoters where mutations or appearing binding sites show properties of interest; or medical data to force the inclusion of certain clinical features or to focus on less-trivial disease markers.

#### Gene expression data analysis

**Fig. 4:**
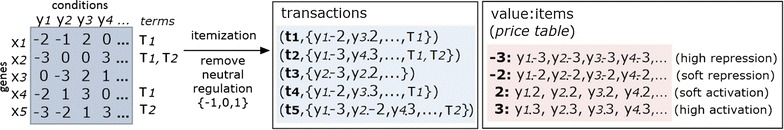
Symbolic dataset and corresponding “price table”

For illustrative purposes, consider Fig. 4 to be associated with a symbolic expression matrix (and associated “price table”), where the rows in the matrix correspond to different genes and their values correspond to the observed expression levels for a specific condition (column). The {−3,−2}, {−1,0,1} and {2,3} sets of symbols are respectively associated with repressed (down-regulated), default (preserved) and activated (up-regulated) expression levels.

First, *succinct* constraints in gene expression analysis allow the discovery of genes with specific constrained levels of expression across a subset of conditions. Illustrating, $min(\varphi _B)$ = −3 implies an interest in biclusters (putative biological processes) where genes are at least highly repressed in one condition. Alternatively, succinct constraints can be used to discover non-trivial biclusters by focusing on non-highly differential expression (e.g. patterns with symbols {−2,2}). Such option contrasts with the large focus on dense biclusters [2], thus enabling the discovery of less-trivial yet coherent modules.

Second, *(anti-)monotone* constraints are key to capture background knowledge and guide biclustering. For instance, the non-succinct monotonic constraint *countVal*$(\varphi _B)\ge 2$ implies that at least two different levels of expression must be present within a bicluster (putative biological process). In gene expression analysis, biclusters should be able to accommodate genes with different ranges of up-regulation and/or down-regulation. Yet, the majority of existing biclustering approaches can only model a single value across conditions [2, 14]. When constraints, such as the value-counting inequality, are available, efficiency bottlenecks can be tackled by adequately pruning the search space.

Finally, *convertible* constraints also play an important role in biological settings to guarantee, for instance, that the observed patterns have an average of values within a specific range. Illustrating, the anti-monotonic convertible constraint $avg(\varphi _B)\le 0$ indicates a preference for patterns with repression mechanisms without a strict exclusion of activation mechanisms. These constraints are useful to focus the discovery on specific expression levels, while still allowing for noise deviations. Understandably, they are a robust alternative to the use of strict bounds from succinct constraints with maximum–minimum inequalities.

#### Biological network data analysis

To motivate the relevance of inputting similar constraints for the analysis of biological networks, we use again the tabular dataset provided in Fig. 4. In this context, rows and columns correspond to nodes associated with biological entities (such as genes, proteins, protein complexes or other molecular compounds), and the values in the matrix correspond to the strength of the interactions between the nodes. As such, the strength of the interactions is either negative {−3, −2} (e.g. inhibition), weak {−1, 0, 1} or positive {2, 3} (e.g. activation).

First, succinct constraints can be specified for the discovery of sets of nodes with specific interaction patterns of interest. Illustrating, $\{-2,2\}\subseteq \varphi _B$ implies an interest on non-dense network modules (coherent interactions with soft inhibition and activation) to disclose non-trivial regulatory activity, and $min(\varphi _B)=-3\wedge max(\varphi _B)=3$ implies a focus on modules with the simultaneous presence of highly positive and negative interactions.

Second, (anti-)monotone constraints are key to discover network modules with distinct yet coherent regulatory interactions. For instance, the non-succinct monotonic constraint *countVal*$(\varphi _B)\ge 3$ implies that at least three different types of interactions must be present within a module.

Finally, convertible constraints are useful to place non-strict expectations on the desirable patterns, yet still accommodating deviations from expectations. Illustrating, $avg(\varphi _B)\le 0$ indicates a preference for network modules with negative interactions without a strict exclusion of positive interactions.

Constraints with nice properties can be alternatively applied for networks with qualitative interactions. Regulatory interactions, such as “binds”, “activates” or “enhances”, are increasingly observed for a wide-variety of protein-protein and gene interaction networks [47, 48]. In this context, assuming the presence of {a, b, c} types of biological interactions, an illustrative anti-monotone constraint is $|\varphi _B\cap \{a,b\}|\ge 0$.

#### Biological data analysis with full-constraints

Although less motivated, constraints can be also defined on the powerset of rows, columns and/or values per columns. In fact, the minimum support and minimum pattern length can be seen as constraints over *I* and *J* indexes, respectively. An alternative constraint over *I* and *J* is to require that biclusters include a minimum number rows/columns from a particular subset of rows/columns of interest. An illustrative succinct constraint in $Y\times \mathcal {L}$ is $P\cap \{y_2$-$3,y_23\}\ne \emptyset$, which implies an interest in biclusters with differential expression (or interactions) associated with the $\mathbf {y}_2$ sample/gene/node.

Please have in mind that the constraints instantiated throughout this section represent a small subset of all possible constraints of interest, thus being mainly introduced for the sake of motivating the relevance of succinct, (anti-)monotone and convertible properties. The specification of constraints of interest is always dependent on the learning goal and the peculiarities of the input data. As such, an exhaustive listing and discussion of relevant constraints for biological data contexts is considered to be out the scope of this work.

### Biclustering with full-constraints

We propose BiClustering with Constraints using PAttern Mining (BiC2PAM) to effectively incorporate full-constraints (including the set of constraints motivated in previous section). BiC2PAM’s extensions to the existing contributions on pattern-based biclustering [12, 14, 19, 20, 22] are twofold. First, a precise formalism was defined to represent full-constraints (with identical notation to the one introduced along this work) and new processing procedures were implemented for their parsing and interpretation. Under these principles, the desirable properties of biclustering solutions can be defined with sharp usability. BiC2PAM supports not only the specification of full-constraints (Definition 10), but further makes available the possibility to specify native constraints to customize the structure, coherency and quality of biclustering solutions (as described in Appendix). Second, BiC2PAM implements different strategies to incorporate distinct types of constraints:

- if native constraints are inputted, BiC2PAM maps them into parameterizations along the mapping, mining and closing steps of BicPAMS (Appendix);
- if constraints without nice properties are inputted, BiC2PAM satisfies them recurring to post-filtering verifications;
- if constraints with nice properties are inputted, BiC2PAM implements pruning heuristics from previous research on constraint-based Apriori-based methods [36, 41].

In the context of the formal view on constraint-based full-pattern mining introduced in "Constraint-based biclustering" section, when constraints over $\Upsilon _P$ (constraints in $2^{\mathcal {L}}$) are inputted, they are mapped as constraints over $P\in 2^{Y\times \mathcal {L}}$. For instance, the $a\in \Upsilon _P$ succinct constraint is mapped as $P\cap \{y_1a,\ldots y_ma\}\ne \emptyset$.

Similarly, constraints from $\psi _P\in 2^{Y}$ are mapped to constraints over $P\in 2^{Y\times \mathcal {L}}$. Illustrating, $y_2\in Y$ is mapped as $P\cap \{y_2a,y_2b,\ldots\}\ne \emptyset$.

Finally, constraints from $\Phi _P\in 2^{X}$ are incorporated by adjusting the Apriori searches to effectively prune the search space. Consider a succinct constraint that specifies a set of transactions to be included in the resulting biclusters. In this case, as soon as a generated candidate is no longer supported by any transaction of interest, there is no need to further generate new candidates and, thus, the search space can be pruned at this point.

Understandably, despite the inherent simplicity of incorporating constraints with nice properties in Apriori-based searches, there is a critical drawback: the inability to rely on key pattern-growth searches, such as F2G (for the discovery of constant/additive/symmetric/plaid biclusters) and IndexSpan (for the discovery of order-preserving biclusters). These pattern-growth searches were previously shown to be able to mine large data with superior efficiency [17, 18]. Adding to this observation, there is a considerable agreement that the underlying structures of pattern-growth searches, such as frequent-pattern trees and prefix-growth trees, provide a more adequate representation of the search space for an improved pruning.

### Exploring efficiency gains from constraints with nice properties

Although the incorporation of constraints with nice properties can only be easily supported under Apriori-based searches, there is large consensus that pattern-growth searches are better positioned to seize efficiency gains from these constraints than peer Apriori-based and vertical searches. As such, F2G-Bonsai and IndexSpanPG, described below, extend respectively the recently proposed F2G (full-frequent itemset miner) and IndexSpan (full-sequential pattern miner) algorithms to guarantee a more effective pruning of the search space in the presence of constraints. These extensions are integrated in BiC2PAM. Native constraints are effectively incorporated in BiC2PAM through adequate parameterizations of pattern-based biclustering algorithms (Appendix).

#### F2G-Bonsai: F2G with itemset constraints

**Fig. 5:**
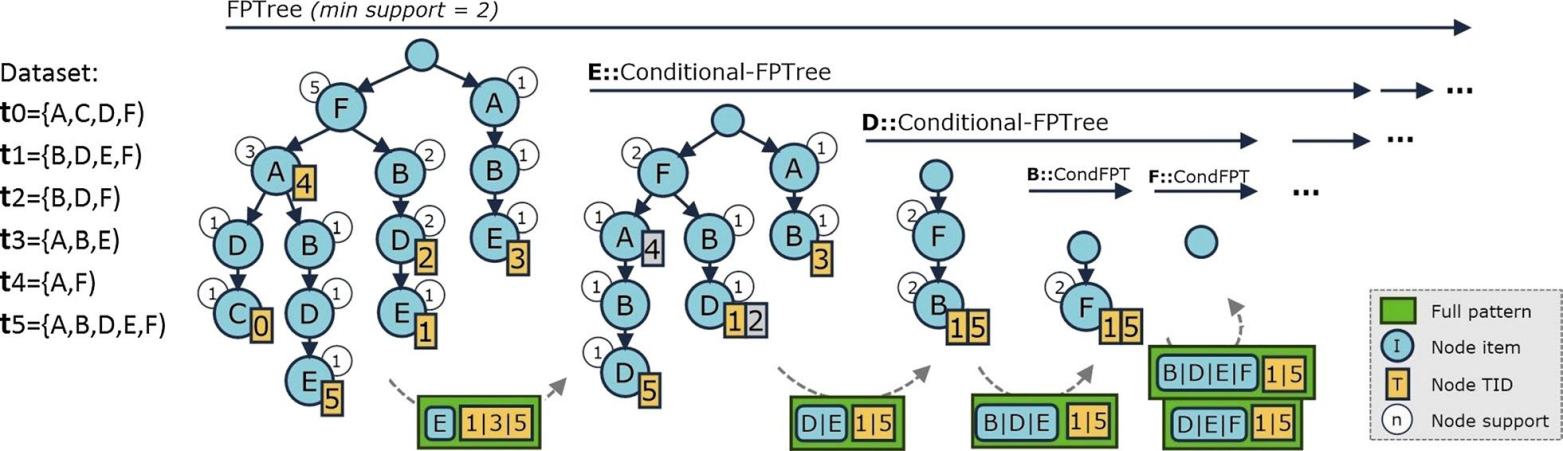
Behavior of F2G (detailed in [17]). The FP-tree is created from the inputted database with transactions annotated in leafs; a conditional pattern is created for each node in the FP-tree; conditional FP-trees are projected from each conditional pattern (transactions moved up along the tree to enable the discovery of full-patterns); conditional FP-trees are recursively mined and patterns grown if frequent; whenever a conditional FP-tree contains a single path, all frequent patterns are enumerated

F2G [17] implements a pattern-growth search that does not suffer from efficiency bottlenecks of peer searches since it relies on frequent pattern tree structures (FP-trees) that store transaction-IDs without duplicates. The FP-tree is efficiently traversed to enumerate all full-patterns. Full-patterns are generated by concatenating the pattern suffixes with the full-patterns discovered from conditional FP-trees where suffixes are removed. Figure 5 instantiates the behavior of F2G. In this section, we first show the compliance of F2G with principles to handle succinct and convertible constraints [15]. Second, we show its compliance to handle difficult combinations of monotone and anti-monotone constraints [33].

##### Compliance with different types of constraints

Unlike candidate generation methods, pattern growth searches provide further pruning opportunities. Pruning principles can be standardly applied on both the original database (FP-Tree) and on each projected database (conditional FP-Tree).

The CFG method extends pattern-growth searches [15] to seize the properties of nice constraints using simplistic principles. Supersets of itemsets violating anti-monotone constraints are removed from each (conditional) FP-Tree. Illustrating, in the presence of $sum(\Upsilon _P)\le 3$, when analyzing the $y_12$ conditional database, the following items $\cup _{i=1}^{m} \{y_i2,y_i3\}$ can be removed to avoid conflicts as their sum violates the given constraint. For an effective pruning, it is recommended to order the symbols in the header table according to their value and support [15, 24]. F2G is compliant with these pruning heuristics, since it allows the rising of transaction-IDs in the FP-Tree according to the order of candidate items for removal in the header table (see Algorithms 1 and 2 in [17]).

For the particular case of an anti-monotone convertible constraint, itemsets that satisfy the constraint are efficiently generated under a pattern-growth search [24]. This is done by assuming that original/conditional FP-trees are built according to a price table and by pruning patterns that no longer satisfy an anti-monotone convertible constraint since the inclusion of new items will no longer satisfy the constraint. Illustrating, since $\{y_1$−$3,y_42,y_23\}$ does not satisfies $avg(\Upsilon _P)\le 0$, there is no need to further build $\{y_1$−$3,y_42,y_23\}$-conditional trees. Therefore, this principle provides an important criterion to stop FP-tree projections and/or prune items in a (conditional) FP-tree.

Finally, the transactions and items within a (conditional) FP-tree that conflict with a given constraint can be directly removed without causing any changes on the resulting set of valid patterns. Illustrating, given $min(\Upsilon _P)=0$ constraint, the transactions $\mathbf {x}_1=\{y_1$−$1,y_23,y_31\}$ and $\mathbf {x}_4=\{y_11,y_2$−$1,y_32\}$ can be directly removed as they do not satisfy this succinct constraint. Similarly, given the same constraint, $min(\Upsilon _P)=0$, the items with values below 0 can be removed. With regards to transactions $\mathbf {x}_1$ and $\mathbf {x}_4$, this means removing $a_{1,1}=y_1$−1 and $a_{4,2}=y_2$−1 items.

Furthermore, constraint checks can be avoided for subsets of itemsets satisfying a monotone constraint. Illustrating, no further checks are needed in the presence of *countVal*$(\Upsilon _P)\ge 2$ constraint when the range of values in the suffix of a pattern is $\ge$2 under the $\{y_10,y_11\}$-conditional FP-Tree.

##### Combination of constraints with nice properties

The previous extensions to pattern-growth searches are not able to effectively comply with monotone constraints when anti-monotone constraints (such as minimum support) are also considered. In FP-Bonsai [33], principles to further explore the monotone properties for pruning the search space are considered without reducing anti-monotone pruning opportunities. This method is based on data-reduction operations originally implemented in ExAnte to seize efficiency gains from the properties of monotone constraints. There are two data-reductions: $\mu$-reduction, which deletes transactions not satisfying *C*; and $\alpha$-reduction, which deletes from transactions single items not satisfying *C*. Thanks to the recursive projections of FP-growth, the ExAnte data-reduction methods can be applied on each conditional FP-tree to obtain a compact number of smaller FP-Trees (FP-Bonsais). The FP-Bonsai method can be combined with the previously introduced principles, which are particularly prone to handle succinct and convertible anti-monotone constraints. F2G can be extended to support these reductions on the (conditional) FP-Trees by guaranteeing that transactions consistently rise up. The only requirement is to preserve the order of items in the header table [17]. As such, F2G complies with the FP-Bonsai extension (see Algorithm 2).

#### IndexSpanPG: IndexSpan with sequential pattern constraints

The work of Pei et al. [27] provides principles to extend pattern-growth searches with prefix-based database projections and no candidate generation to effectively incorporate regular expressions and constraints with nice properties. For this aim, the prefix-monotone property is defined. A constraint is called *prefix-monotone* if it is prefix anti-monotonic or prefix monotonic. With a prefix-monotone constraint, there is only the need to search in the projected databases for prefixes that satisfy the constraint. When a constraint *C* is: (1) prefix anti-monotonic, if *C*(*P*) = false, then there exists no sequential patterns containing *P* has a prefix and also satisfies *C*; (2) prefix monotonic, if *C*(*P*) = true, then every sequential pattern having *P* as a prefix satisfies *C*; and (3) a regular expression, if the prefix of a given sequential pattern is conflicting with the regular expression *C*, then there is no need to further expand (i.e. there are no sequential patterns with the same prefix that also satisfy *C*). As such, since monotonic, anti-monotonic and regular expression constraints are prefix-monotone they can be pushed deep into the search. Understandably, the efficiency gains associated with such constraints cannot be attained under Apriori-based searches [41]. Although succinct constraints are not necessarily prefix anti-monotonic or prefix monotonic, they can also be easily pushed deep into the mining process (independently of the applied SPM method).

According to these principles, we extended IndexSpan [18], an extension of PrefixSpan to explore efficiency gains from the intrinsic properties of the order-preserving biclustering task. IndexSpan is compliant with the enumerated principles. The minimalist data structures, fast database projections and early pruning techniques [18] do not interfere with the underlying prefix-growth behavior, the essential requirement to incorporate prefix-monotone constraints. Furthermore, given the fact that IndexSpan explores item-indexable properties associated with the order-preserving biclustering task, testing constraints is done in an efficient and elegant way (see Algorithm 3). This is true with regards to both: (1) the validation of whether an anti-monotonic constraint (or regular expression) cannot be satisfied by a given prefix (in order to stop its growth), and (2) the validation of whether a a monotonic constraint cannot be satisfied by a given (projected) sequence (in order to prune the search).

### BiC2PAM: algorithmic details

**Fig. 6:**
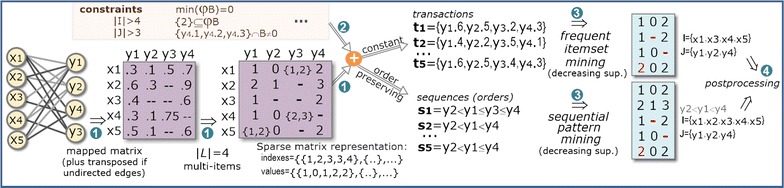
Simplified illustration of BiC2PAM behavior: (*1*) transactional and sequential databases are derived from a multi-item matrix; (*2*) constraints are processed; (*3*) pattern mining searches are applied with a decreasing support; and (*4*) the discovered pattern-based biclusters that satisfy the inputted constraints are postprocessed

The algorithmic basis of BiC2PAM is described in Algorithm 1. The behavior of BiC2PAM can be divided according to four major steps: (1) preprocessing, (2) instantiation of constraints, (3) mining and (4) postprocessing. In step 1, the input real-valued matrix is discretized (after proper normalization and exclusion of outliers) under a given coherency strength, and multiple items assign to values near a boundary of discretization (according to [14]). If, instead, a network is given as input, it is mapped into a sparse adjacency matrix (according to [3]). Still along this first step, transactional and sequential databases are mapped from the previous data structures. In step 2, the inputted constraints are parsed, their soundness checked against the preprocessed databases, and used to parameterize BiC2PAM (if native) or instantiated (otherwise). In step 3, the pattern mining searches proposed in "Exploring efficiency gains from constraints with nice properties" section are applied over the mapped databases and inputted constraints with a decreasing support until a pre-specified number of pattern-based biclusters (or coverage of matrix elements) satisfying these constraints is achieved. Finally, BiC2PAM allows for the postprocessing of the discovered biclusters to guarantee their robustness and dissimilarity by recurring to merging, extension, reduction and filtering procedures (step 4 according to [14]). Figure 6 provides a simplified illustration of these major steps.

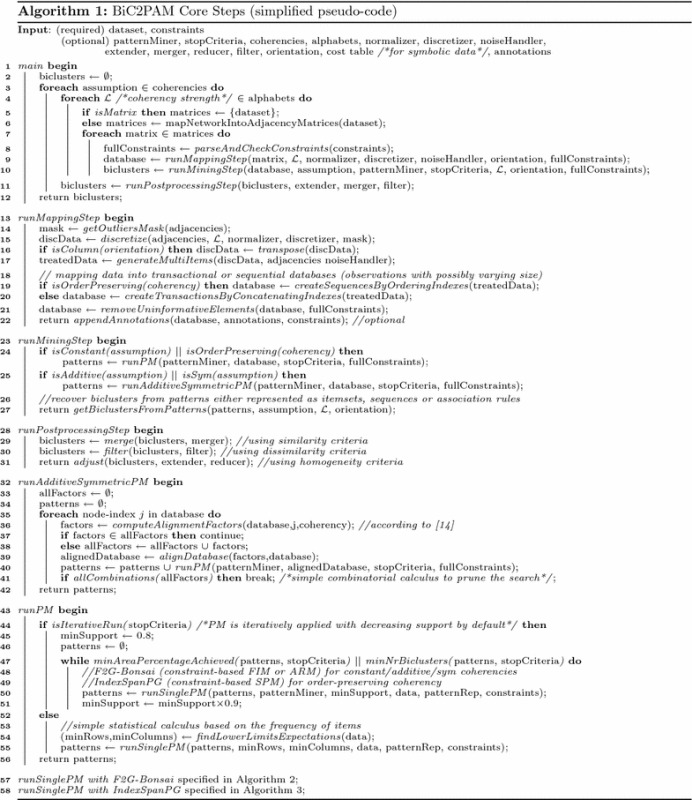

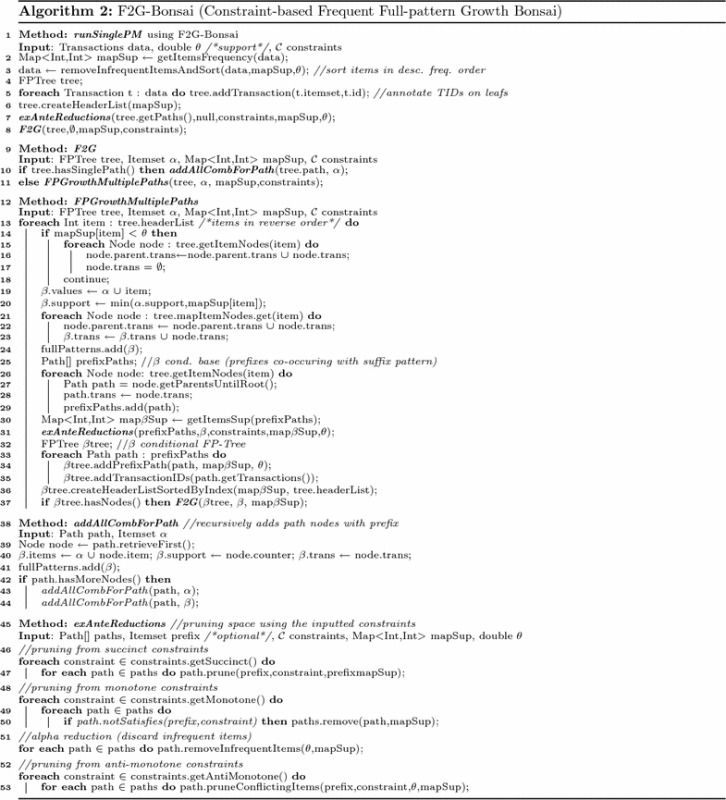





Understandably, the behavior and performance of Algorithm 1 is essentially dependent on the underlying domain-driven pattern mining searches. Algorithms 2 and 3 respectively describe F2G-Bonsai and IndexSpanPG in accordance with the pruning principles respectively introduced in "F2G-Bonsai: F2G with itemset constraints" and "IndexSpanPG: indexSpan with sequential pattern constraints" sections. In F2G-Bonsai, reductions of the search space are efficiently applied during the creation of the initial FP-tree and of each conditional FP-tree (lines 7 and 32). Succinct, monotone, frequency and anti-monotone reductions are efficiently applied in this order. In IndexSpanPG, the pruning of conflicting sequences or items with sequential constraints is done after the initial construction of the item-indexable database and after each database projection (lines 6, 24 and 29). Moreover, the growing of a given prefix is stopped whenever the prefix contradicts an anti-monotonic constraint or regular expression (lines 21 and 26). In order to avoid an unnecessary overhead for biclustering tasks in the presence of high number of constraints, the pruning principles in F2G-Bonsai and IndexSpanPG might be only applied for certain database projections. In this case, the periodicity $\tau$ of projections eligible for pruning should be given as input to the algorithms ($\tau$ = 1 by default).

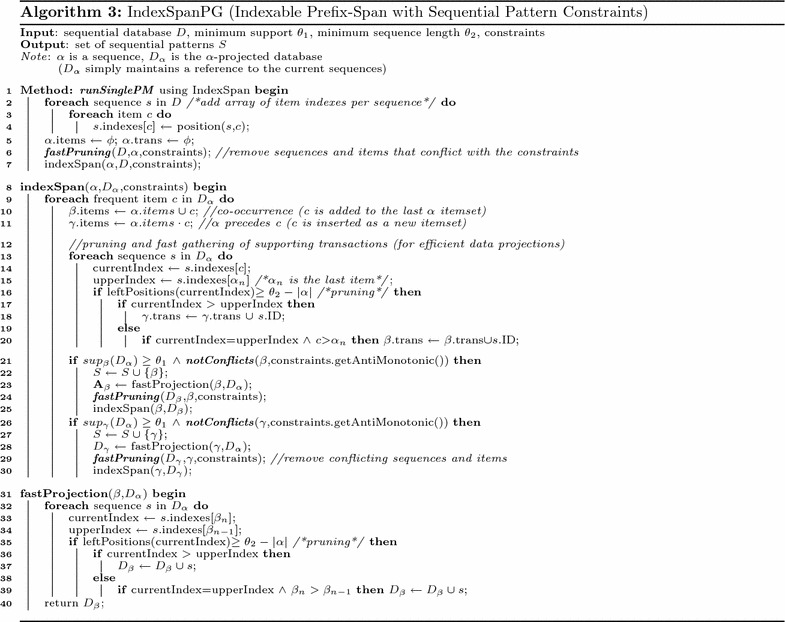



The computational complexity of BiC2PAM is bounded by the complexity of the pattern-based biclustering task in the absence of constraints. The complexity of pattern-based biclustering tasks for dense and sparse matrices can be respectively consulted in the documentation of BicPAM [14] and BicNET [3].

BiC2PAM also provides default behaviors in order to guarantee a friendly environment for users without expertise in biclustering. For this aim, BiC2PAM makes available: (1) default parameterizations (data-independent setting) and (2) dynamic parameterizations (data-dependent setting). Default parameterizations include: (1) zero-mean row-oriented normalization followed by overall Gaussian discretization with *n*/4 items for order-preserving coherencies (for an adequate trade-off of precedences vs. co-occurrences) and a set of $\{3,5,7\}$ items for the remaining coherencies; (2) iterative discovery of biclusters with distinct coherencies (constant, symmetric, additive and order-preserving); (3) F2G-Bonsai search for closed FIM and association rule mining, and IndexSpanPG search for SPM; (4) multi-item assignments; (5) merging of biclusters with over 70 % Jaccard-based similarity; (6) a filtering procedure for biclusters without statistical significance (according to [49]) and a 60 % Jaccard-based similarity against a larger bicluster; and (7) no constraints. For the default setting, BiC2PAM iteratively decreases the support threshold by 10 % (starting with $\theta$ = 80 %) until the output solution discovers 50 dissimilar biclusters or a minimum coverage of 10 % of the inputted matrix elements or network interactions. Dynamic parameterizations enable the: (1) selection of data-driven normalization and discretization procedures according to their fitting error, and (2) activation of data partitioning procedures for large matrices: over 100 million elements (excluding missing values) for the discovery of constant biclusters and over 1 million elements for the remaining coherencies.

## Results

This section provides empirical evidence of the soundness of the proposed contributions and of the relevance of using constraints within (pattern-based) biclustering to prune the search space and guarantee biologically significant solutions. To this end, we assessed the performance of BiC2PAM on synthetic data, gene expression data and biological networks in the presence of domain knowledge. BiC2PAM was parameterized with default behavior and applied with F2G-Bonsai for the discovery of constant biclusters with itemset constraints and with IndexSpanPG for the discovery of order-preserving biclusters with sequential pattern constraints. The stopping criteria of BiC2PAM was specified as a minimum of 20 dissimilar biclusters for synthetic data contexts and 50 dissimilar biclusters for real data contexts. BiC2PAM is implemented in Java (JVM v1.6.0-24). The experiments were computed using an Intel Core i5 2.30GHz with 6GB of RAM.

### Results on synthetic data

#### Synthetic data

Table 1 describes the generated data settings, with properties resembling the regularities of gene expression data. Constant and order-preserving biclusters with varying quality and coherency strength were generated. Noise factors (±20 % of the range of inputted values) were imputed and overlaps between biclusters allowed. The selected number of rows and columns per bicluster follows a Uniform distribution using the ranges in Table 1 in order to guarantee the inclusion of biclusters with dissimilar shapes. Reported results are the average of performance views collected from 30 data instances per setting.

**Table 1:** Properties of the generated dataset settings.

Non-exhaustive list of matrices ($\sharp$rows $\times$ $\sharp$columns)	500 × 50	1000 × 100	2000 × 200	4000 × 400
Number of hidden biclusters (*K*)	$6\times \frac{1}{\mu }$	$10\times \frac{1}{\mu }$	$15\times \frac{1}{\mu }$	$20\times \frac{1}{\mu }$
Number of rows per hidden bicluster	$\mu$[50,70]	$\mu$[70,100]	$\mu$[100,200]	$\mu$[200,300]
Number of columns per hidden bicluster	$\mu$[5,7]	$\mu$[7,10]	$\mu$[8,12]	$\mu$[10,15]

where $\mu$ defines the flexibility of the underlying coherency assumption ($\mu$ = 1 for constant and $\mu$ = 2 for order-preserving)

Additional properties (default settings in bold):

Coherency strength $\delta$ = {5, 10, 15, **20**, 25, 33 %} (or symbols $|\mathcal {L}|$ = {20, 10, 7, **5**, 4, 3})

Deviations on data values in {0, $\varvec{\delta }$/2, ${\delta }$, 2$\delta$}, and degree of noisy and missing elements in {0, **2**, 5, 10 %}

Overlapping degree $\theta$ = {0, 0.1, **0.2**, 0.4} with plaid effects$^2$ described by *f* = {**sum**, product, weighted} (cumulative function) $\nu$ = {**1**, 0.7, 0.4} (cumulative effect), $\epsilon$ = {**0.1**, 0.2} (noise), $\kappa$ = {0.5, **0.3**, 0.1 K} (average number of interacting biclusters) and $\phi$ = {1, **0.8**, 0.5} (distribution of overlapping areas between the $\kappa$ bics)— variables according to [20]

#### Uninformative elements

**Fig. 7:**
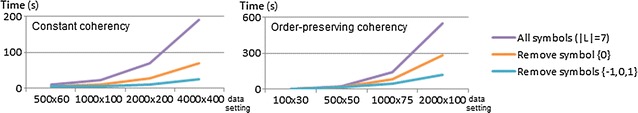
Efficiency gains of BiC2PAM from succinct constraints specifying uninformative elements for varying data settings with constant and order-preserving biclusters and coherency strength defined by $|\mathcal {L}|$ = 7

A simplistic yet relevant form of domain knowledge is the knowledge regarding the uninformative elements of a given dataset. To this end, the ranges of values (or symbols) to remove can be specified under a succinct constraint $S\notin P$ where $S\subseteq \mathbb {R}^+$ (or $S\subseteq \mathcal {L}$). The application of this constraint within BiC2PAM leads to the removal of these elements prior to the mining step, resulting in significantly large efficiency gains as shown by Fig. 7. This figure describes the impact of removing a varying extent of uninformative elements from synthetic data on the biclustering task. Despite the simplicity of this constraint, existing biclustering algorithms are not able to support this behavior, which undesirably impacts their efficiency and the adequacy of the outputted biclustering solutions.

#### Incorporating annotations

**Fig. 8:**
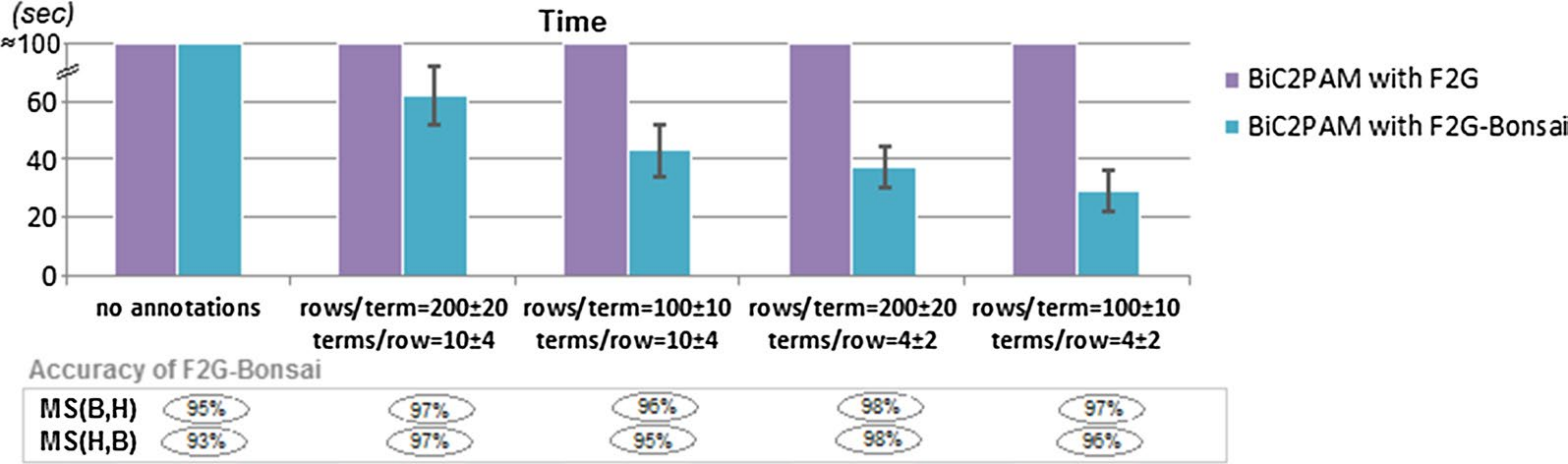
BiC2PAM ability to biclustering data with varying distributions of annotations (efficiency and Jaccard-based match scores [14] collected for the 2000 × 200 setting)

Figure 8 assesses the ability of BiC2PAM to discover biclusters with functional consistency from annotated data. Functional consistency is observed when the majority of rows in a bicluster share one or more annotations. To this end, we annotate 2000 × 200 matrices with a varying number of annotations per row^2^, {10 ± 4, 4 ± 2}, where each annotation is observed on a varying number of rows, {200 ± 10, 100 ± 10}. For this analysis, we guaranteed that the hidden biclusters have a high degree of functional consistency by imposing that the majority (85 % ± 10 pp) of their rows share a common annotation. As such, BiC2PAM was parameterized with succinct constraints guaranteeing that at least one annotation is consistently observed for all the rows of each bicluster before postprocessing (before the application of extension, merging and reduction procedures). Despite the higher complexity from mining heterogeneous data (input data plus a large amount of annotations), results show that BiC2PAM is in fact more efficient than the baseline option. Furthermore, the observed match scores suggests that the presence of annotations may play an important role in guiding the recovery of true biclusters.

#### Itemset constraints

**Fig. 9:**
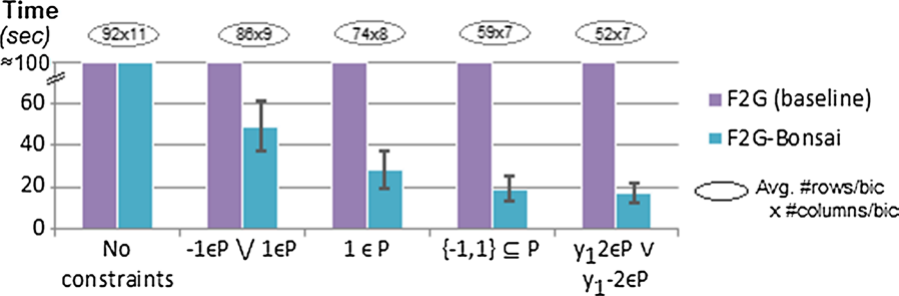
BiC2PAM’s efficiency in the presence of succinct constraints (2000 × 200 setting with constant assumption)

In order to test the ability of BiC2PAM to seize efficiency gains in the presence of itemset constraints with nice properties, we applied BiC2PAM over the 2000 × 200 data setting (generated with 5 background symbols $$\mathcal {L}$$ = {−2, −1, 0, 1, 2} and hidden biclusters with constant assumption) in the presence of succinct, monotone and convertible constraints. For the baseline performance, constraints were satisfied using post-filtering procedures. Figure 9 shows the impact of inputting disjunctions of succinct constraints in the performance of BiC2PAM. As observed, the ability of BiC2PAM to effectively prune the search space in the presence of these constraints is associated with significant efficiency gains. Moreover, they enable a focus on less-trivial regions from the input data space (e.g. −1 $$\in$$$$\varphi _B\vee 1$$$$\in$$$$\varphi _B$$).

**Fig. 10:**
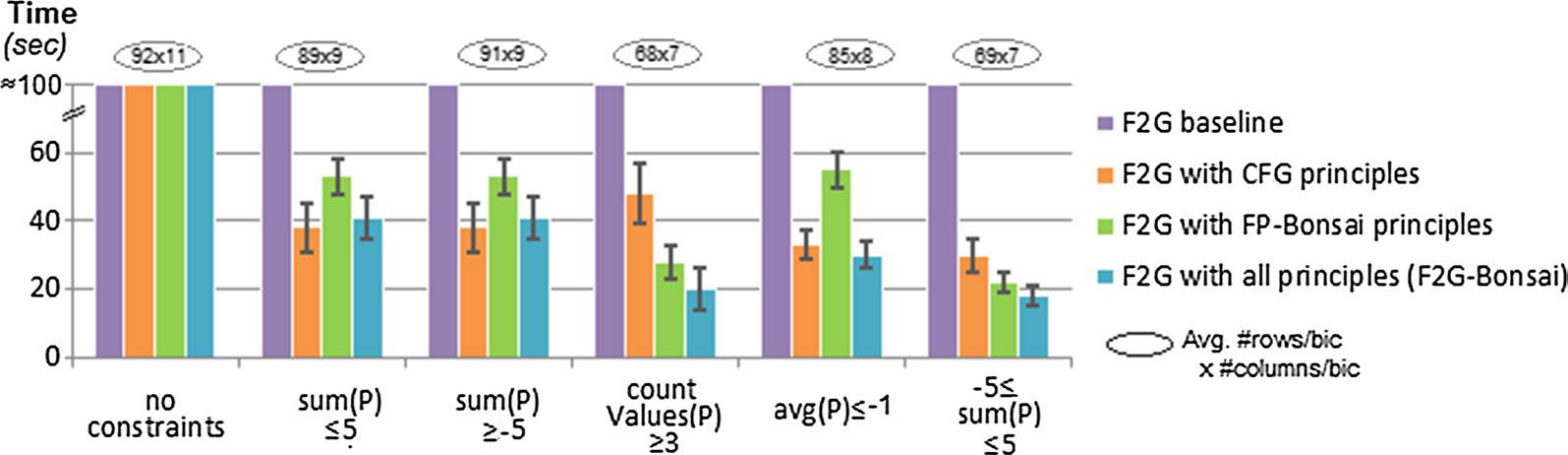
BiC2PAM’s efficiency with (combined) anti-monotone, monotone and convertible constraints (2000 × 200 setting with constant coherency). Impact of enhancing BiC2PAM with CFG [15] and FP-Bonsai [33] principles

Figure 10 measures the performance of BiC2PAM when constraints with monotone, anti-monotone and convertible properties are inputted. To this end, we show the efficiency gains from parameterizing the underlying F2G miner with diverse principles, and further test F2G’s ability to deal not only with constraints satisfying a single property but multiple properties of interests (e.g. $\gamma _1<sum(\varphi _B)<\gamma _2$). Results confirm that the proposed enhancements can lead to a substantial pruning of the search space. In particular, CFG principles [15] are used to seize efficiency gains from convertible constraints and FP-Bonsai [33] to seize efficiency gains from monotonic constraints.

#### Sequential pattern constraints

**Fig. 11:**
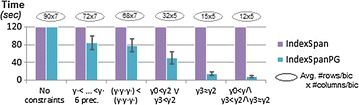
BiC2PAM performance with sequence constraints when learning order-preserving solutions (1000 × 100 setting)

Figure 11 extends the previous analyses towards the constraint-guided discovery of order-preserving biclusters with regular expressions. For this analysis, BiC2PAM was parameterized with IndexSpan and IndexSpanPG and applied over the 1000 × 100 setting with a varying set of constraints (minimum number of precedences and ordering constraints). Results show that increased efficiency gains can be attained from pruning data regions that do not satisfy these constraints.

#### Full-pattern growth searches

**Fig. 12:**
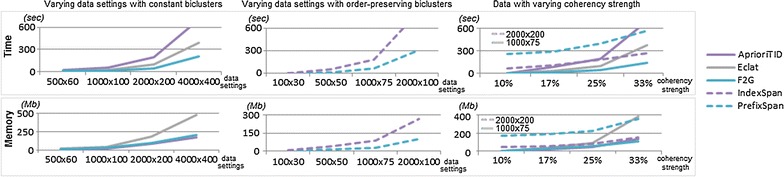
Impact of full-pattern growth searches in the performance of BiC2PAM for data with varying size (under a fixed coherency strength $\delta$ = 20 %) and for fixed data settings with varying coherency strength

The previous results highlight the relevance of full-pattern growth searches for biclustering (F2G-Bonsai and IndexSpanPG) to adequately prune the search space. Figure 12 further motivates the importance of the proposed F2G-Bonsai against AprioriTID and Eclat (F2G is able to surpass efficiency bottlenecks associated with bitset data structures), and the relevance of IndexSpanPG against PrefixSpan (IndexSpan is able to explore further efficiency gains from the item-indexable properties of the biclustering task). Results show the relevance of parameterizing BiC2PAM with the proposed full-pattern growth searches for large data and for hidden biclusters with loose coherency strength (highly dense data).

### Results on biological data

#### Real data

To assess BiC2PAM over real data, we selected expression and network datasets with varying properties. Four gene expression datasets were considered: *dlblc* (660 genes, 180 conditions) with human responses to chemotherapy [50], *hughes* (6300 genes, 300 conditions) to study nucleosome occupancy [51], and *yeast-cycle* (6221 genes, 80 conditions) and *gasch* (6152 genes, 176 conditions) measuring yeast responses to environmental stimuli [52]. Three biological networks from STRING v10 database [53] were additionally considered. These networks capture the gene interactions within human (6314 nodes, 423,335 interactions),* Escherichia coli* (8428 nodes, 3,293,416 interactions) and yeast (19,247 nodes, 8,548,002 interactions) organisms. The scores in these networks are inferred from literature and multiple data sources, revealing the expected strength of correlation between genes.

**Fig. 13:**
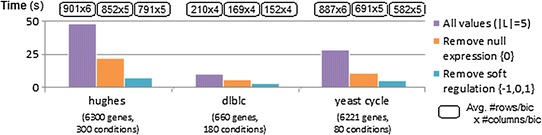
Efficiency of BiC2PAM with knowledge regarding the uninformative elements for the analysis of expression data (hughes, dlblc, yeast-cycle) when assuming a constant coherency with $|\mathcal {L}|$  = 5

#### Uninformative elements

**Fig. 14:**
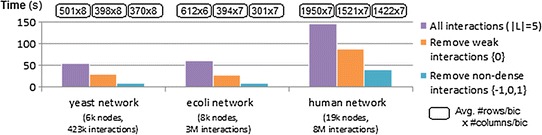
Efficiency of BiC2PAM with knowledge regarding the uninformative elements for the analysis of network data (human,* Escherichia coli*, yeast from STRING [53]) when assuming constant coherency with $|\mathcal {L}|$ = 5

In gene expression data analysis, elements from the input matrix with default/non-differential expression are generally less relevant. Similarly, in the context of network data analysis, interactions with low weights are generally of reduced interest for module discovery. In these contexts, these data elements can be removed from the learning under a succinct constraint. Figures 13 and 14 measures the impact of inputting such succinct constraints on the efficiency of BiC2PAM and on the properties of the outputted biclusters (assuming constant coherency). For this analysis, we analyze performance of BiC2PAM on both expression data (Fig. 13) and network data (Fig. 14) from different organisms. Results show that by inputting such simplistic constraints, very high efficiency gains can be obtained. Additionally, the removal of uninformative elements allows the focus on more relevant regions of the input data space and is associated with slightly smaller biclusters due to the greater ability to exclude such elements from the solution space.

#### Annotations

**Fig. 15:**
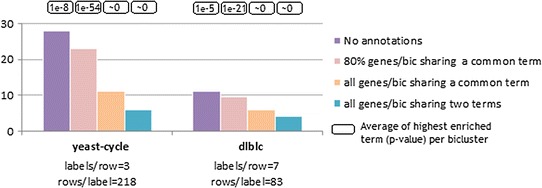
Performance of BiC2PAM for biclustering biological datasets (yeast-cycle and dlblc) annotated with representative human and yeast GO terms (terms associated with biological processes with more than 50 genes)

Figure 15 measures the impact of incorporating functional terms from ontologies for the analysis of biological data (assuming an underlying constant coherency). To this end, we collected for each gene from human and yeast organisms the set of functional terms associated with the biological processes represented in gene ontology from GOToolBox [46]. BiC2PAM was then applied over expression and network data in the presence of these annotations. Results confirm that BiC2PAM is able to integratively learn from data and annotations without further costs in efficiency, and to guarantee the functional consistency of the outputted biclusters (as expectedly demonstrated by the analysis of the enriched terms).

#### Succinct, monotone and convertible constraints

**Fig. 16:**
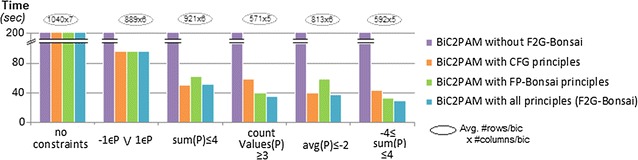
Efficiency gains from using biologically meaningful constraints with succinct/monotone/convertible properties within BiC2PAM for the analysis of the gasch dataset (6152 × 176)

**Fig. 17:**
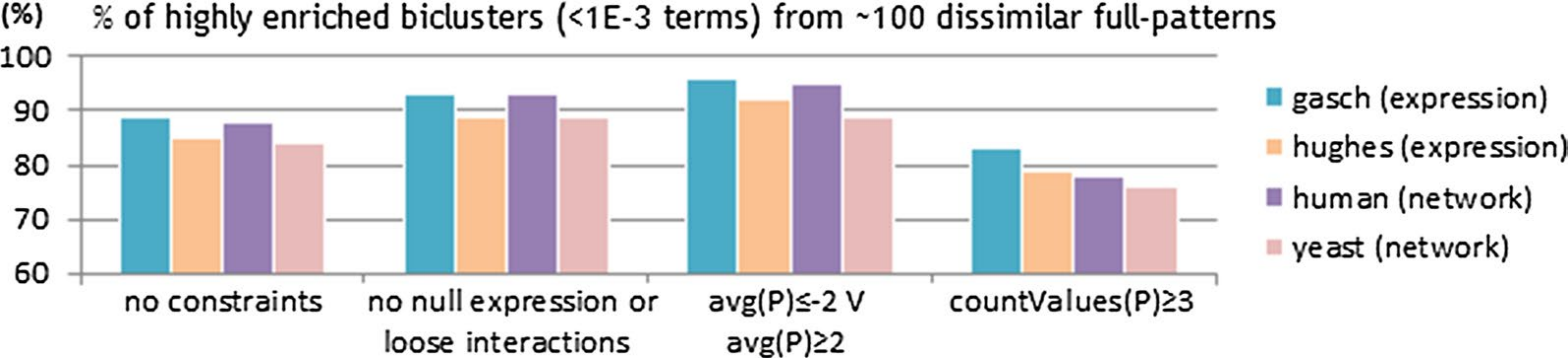
Biological relevance of BiC2PAM for different constraint-based profiles of expression

Figures 16 and 17 show the impact of inputting biologically meaningful constraints in the efficiency and effectiveness of BiC2PAM. For this purpose, we used the complete gasch dataset (6152 × 176) [54] with five levels of expression ($|\mathcal {L}|$ = 6). The impact of considering a diverse set of constraints in the efficiency levels of BiC2PAM is provided in Fig. 16. The observed results demonstrate the relevance of using meaningful constraints with succinct, (anti-)monotone and convertible properties not only to guarantee a user-guided focus on specific regions of interest, but also to promote the tractability to perform biclustering to solve computationally complex biological problems and analyzes.

The impact of these constraints in the relevance of pattern-based biclustering solutions is presented in Fig. 17. The biological relevance of each bicluster was derived from the analysis of functionally enriched GO terms based on the application of hypergeometric tests [46]. A bicluster is considered significantly enriched if it has a set of correlated over-represented terms with Bonferroni corrected *p* values below $10^{-3}$. Two major observations can be retrieved. First, when focusing on properties of interest (e.g. differential expression), the average significance of biclusters increases as their genes have higher propensity to be functionally co-regulated. This trend is observed despite the smaller size of the constrained biclusters. Second, when focusing on rare expression profiles ($\ge$3 distinct levels of expression), the average relevance of biclusters slightly decreases as their co-regulation is less obvious. Yet, such non-trivial biclusters hold unique properties with potential interest that can be further investigated. To our knowledge, BiC2PAM is the only available biclustering algorithm able to rely on user expectations and other forms of knowledge to focus the search on these non-trivial yet coherent and potentially interesting regions from the input data space.

## Conclusions and future work

This work motivates the relevance of constraint-guided biclustering for biological data analysis with domain knowledge. To answer this task, we explored the synergies between pattern-based biclustering and domain-driven pattern mining. As a result, BiC2PAM algorithm was proposed with two major goals: (1) to learn biclustering models in the presence of an arbitrary number of annotations from knowledge repositories and literature, and (2) to effectively incorporate constraints with nice properties derived from user expectations. BiC2PAM can therefore be applied in the presence of domain knowledge to guarantee a focus on relevant regions and explore potentially high efficiency gains.

We further demonstrated the consistency between domain-driven pattern mining and pattern-based biclustering based on the notion of full-patterns; surveyed the major drawbacks of existing research towards this end; and extended pattern-growth searches with state-of-the-art principles to prune the search space by pushing constraints with nice properties deep into the mining process. In particular, we showed the compliance of F2G searches with principles to effectively prune (conditional) FP-Trees, and the compliance of IndexSpan searches with principles to effectively prune prefix-growth structures. These searches were respectively extended to support pattern-based biclustering with constant and order-preserving assumptions.

Meaningful constraints with succinct, monotone, anti-monotone and convertible properties were presented for distinct biological tasks (gene expression analysis and network data analysis) in order to focus the search space on less-trivial yet coherent regions.

Results from synthetic and real data show that the incorporation of background knowledge leads to large efficiency gains that turn the biclustering task tractable for large-scale data. We further provide initial evidence of the relevance of the supported types of constraints to discover non-trivial yet meaningful biclusters in expression and network data with heightened biological significance.

Four major directions are identified for future work. First, the extension of the proposed contributions towards classification tasks based on the discriminative properties of biclusters in labeled data contexts. Second, an in-depth systematization of constraints with nice properties across biological data domains, including a structured view on their relevance for omic, genome-wide and chemical data analysis. Third, a broader quantification of the impact of incorporating constraints across these data domains. Finally, the extension of the proposed framework for the tasks of biclustering time series data and triclustering multivariate time series data in the presence of temporal constraints.

## Data and software availability

The datasets and BiC2PAM software are available in http://web.ist.utl.pt/rmch/software/bic2pam/.

## Appendix: Native constraints

In addition to the incorporation of functional annotations and specification of constraints with properties of interest, further possibilities can be explored within BiC2PAM to guarantee its ability to learn biclustering solutions with customizable structure, coherency and quality in accordance with domain knowledge. Below we list a set of native constraints to this end that are effectively incorporated within BiC2PAM by adapting the parameters that control its behavior along its preprocessing, mining, postprocessing steps.

Relevant constraints provided in the preprocessing step include:

- Minimum coherency strength of the target biclusters (Definition 2). Decreasing the coherency strength (increasing the number of symbols) reduces the allowed deviations from value expectations and it is often associated with solutions composed by a higher number of smaller biclusters;
- Tolerance to noise $\eta _{ij}$ (Definition 2). This constraint is used to adjust the behavior of BiC2PAM in the presence of noise, missing values or discretization drawbacks. BiC2PAM enables the possibility to assign a parameterizable number of symbols to a given data element when its value is near a boundary of discretization. By assigning two or more symbols guarantees a higher robustness to noise (proof in [14]).

Relevant constraints provided in the mining step include:

- Coherency assumption and orientation: Currently, BiC2PAM supports the selection of constant, additive, multiplicative, symmetric, order-preserving and plaid models with coherency on rows or columns. An in-depth view on the relevance of non-constant coherency assumptions for expression and network data analysis was previously provided in [14, 19, 20, 22].
- Minimum pattern length and/or support (minimum number of columns and/or rows in the bicluster).
- Pattern representation: simple (all coherent biclusters), closed (all maximal biclusters), or maximal (solutions with a compact number of biclusters with a preference towards a high number of columns).
- Stopping criteria: minimum number of biclusters able to satisfy the inputted constraints, or minimum area of the input matrix covered by the discovered valid biclusters.

Understandably, constraints addressed at the postprocessing stage are not desirable since they are not able to seize major efficiency gains. Nevertheless, BiC2PAM supports three key types of constraints that could imply additional computational costs, but are addressed with heightened efficiency: (1) maximum percentage of noisy and missing elements per bicluster (based on merging procedures [14]), (2) minimum homogeneity of the target biclusters (using extension and reduction procedures with a parameterizable merit function [14]) and (3) minimum dissimilarity criteria to guarantee compact outputs.

Previous work from Henriques and Madeira [1, 14, 19, 20, 22] provide an in-depth description of how pattern-based biclustering algorithms implement this wide-set of customization possibilities.

The listed native constraints can be specified in declarative form. As such, BiC2PAM provides the possibility to affect structural aspects of its outputs with sharp usability.

## Abbreviations

BicNET: Biclustering NETworks (algorithm)
Bic2PAM: BiClustering with Constraints using PAttern Mining (algorithm)
BicPAM: BiClustering using PAttern Mining (algorithm)
BicSPAM: Biclustering using Sequential PAttern Mining (algorithm)
BiModule: Biclustering Modules (algorithm)
BiP: Biclustering Plaid models (algorithm)
DeBi: Differentially expressed Biclustering (algorithm)
F2G: Full Frequent-pattern Growth
FIM: Frequent Itemset Mining
FP: Frequent Pattern
GO: Gene Ontology
SPM: Sequential Pattern Mining

## Footnotes

1 Biclustering involves combinatorial optimization to select and group rows and columns and it is known to be a NP-hard problem (proven by mapping the problem of finding maximum edge (bi)clique in a bipartite graph into the problem of finding dense biclusters with maximum size [2, 10]). The problem complexity increases for non-binary data contexts and when elements are allowed to participate in more than one bicluster (non-exclusive structure) and in no bicluster at all (non-exhaustive structure).

2 Datasets available in http://web.ist.utl.pt/rmch/software/bic2pam/.
